# Prevalence and Correlates of Mental Health of University Students in Hong Kong: What Happened One Year After the Occurrence of COVID-19?

**DOI:** 10.3389/fpubh.2022.857147

**Published:** 2022-06-29

**Authors:** Daniel T. L. Shek, Diya Dou, Xiaoqin Zhu

**Affiliations:** Department of Applied Social Sciences, The Hong Kong Polytechnic University, Kowloon, Hong Kong SAR, China

**Keywords:** DASS, anxiety, depression, stress, University students, economic strain, living alone

## Abstract

**Purpose:**

In this study, we studied the prevalence and correlates of mental health problems (depression, anxiety, and stress) in Hong Kong university students after one year of the occurrence of COVID-19. We examined the relationships between mental health problems and socio-demographic factors (including age, gender, local/international students, living status, and economic strain), need satisfaction, and difficulties encountered.

**Methods:**

A cross-sectional online survey involving 1,648 university students (Mean age = 20.09 years ± 1.37) was conducted. They responded to a questionnaire assessing their mental health (including the Depression, Anxiety, and Stress Scales—DASS), psychosocial resources, social support, need satisfaction, difficulties and challenges, and evaluation of services they received.

**Results:**

Based on DASS cutoff scores, prevalence rates of moderate or above levels of depression, anxiety, and stress were 40.0, 50.7, and 22.2%, respectively. While age and gender were not related to the DASS measures, economic strain and living alone were positively related to negative emotional states based on the results of MANOVAs. Multiple regression and PROCESS analyses showed that need satisfaction and difficulties contributed to depression and anxiety, with stress as a mediating factor.

**Conclusions:**

The proportions of students who experienced moderate and above levels of depression (40.0%), anxiety (50.7%), and stress (22.2%) indexed by DASS deserve attention. Living alone and experienced economic disadvantage are risk factors for negative emotional states. Need satisfaction and life challenges are related to negative emotional states. Need satisfaction and difficulties contributed to stress, which further influenced anxiety and depression.

## Introduction

The COVID-19 pandemic has created many global impacts. Since December 2019, the total deaths have been more than 6 million (1), and many societies have experienced lockdown and other social distancing measures. Amongst different age groups, university students are much affected by COVID-19 for several reasons. First, university students must adjust to the change from a face-to-face learning mode to an online one. The adjustment would not be easy, particularly for those experiencing economic strains such as having limited computer and Wi-Fi resources. Second, because of city and school lockdown, some students have difficulties in traveling from student hostels to home (i.e., forced hostel stay) or from home back to student hostels (i.e., forced homestay), which adversely affects their social life and support network. Third, it is common for university students to take up part-time work to support their living. Unfortunately, it is harder for them to get appropriate part-time jobs than before because of the city lockdown and economic slowdown during COVID-19. Thus, university students may encounter financial difficulties. Finally, as there is the myth that university students are “adults” who can take care of themselves, their problems may be overlooked. Shek and Wong (2) argued that educators commonly had the misconception that adolescents' problems would “disappear overnight” after they entered universities.

There are studies investigating the adjustment and mental health of college students under COVID 19. Essadek and Rabeyron (3) identified that around 40% of French students suffered from distress, anxiety, and depression, and 15% even had thoughts of self-harming. An increase in stress and anxiety levels among university students during the pandemic was reported in the US (4), France (5), Spain (6), and Poland (7). Similarly, surveys conducted in mainland China, Japan, South Korea, and Indonesia also showed an increase in the number of students suffering from negative emotions and mental health problems during COVID-19 (8–11). A recent meta-analytic review involving 84 studies in mainland China found an overall prevalence rate of 26% of depressive symptoms among university students and 1.69% displayed severe psychological symptoms (11).

Interestingly, although many studies have examined the mental health of university students in mainland China, comparatively fewer studies have examined the mental health of university students in Hong Kong under COVID-19. There are several reasons why findings based on university students in Hong Kong should be collected. First, earlier studies suggested that mental health issues among university students in Hong Kong were disturbing. Li et al. (12) showed that 32.7, 44.7, and 18.5% of the university students could be regarded as having moderate or above levels of depression, anxiety, and distress, respectively. They also found that gender, wellbeing, and political events were related to student mental health. Second, the mental health issues of young people in Hong Kong may be further intensified by the social unrest from 2019 to 2020 (13). In fact, Shek and Siu (14) argued that young people in Hong Kong grew up in an “unhappy” environment. With such a backdrop, COVID-19 may exert additional psychological burdens on young people in Hong Kong (15). Third, as there are divergent views on the strategies to cope with COVID-19 (such as the degree of lockdown in early 2020) and politics involving COVID-19 (such as calling COVID-19 as “Wuhan pneumonia,” assuming that the disease originated from Wuhan and China should be responsible for the global pandemic), COVID-19 constitutes a stressor for people in Hong Kong.

Shek (16) proposed that different ecological factors might influence the mental health status of university students under COVID-19. Primarily, experiences of university students in the “personal” system under COVID-19 are of paramount importance, such as how well one's personal needs are satisfied during COVID-19. According to the self-determination theory (17), whether individual needs are met would influence individual adjustment. While gratifying basic physiological and psychological needs promotes positive wellbeing, need frustration would lead to mental health problems. This notion is supported by research findings in times of COVID-19: Schutte and Malouff (18) showed that need satisfaction was positively associated with positive affect and better mental health; Šakan et al. (19) reported that need satisfaction positively predicted wellbeing under the pandemic. Nevertheless, research on need satisfaction has commonly focused on basic psychological needs (i.e., autonomy, competence, and relatedness) instead of personal needs in the specific context of COVID-19. The abrupt changes in daily routines under COVID-19 due to city lockdown and other social distancing measures such as restriction of social gathering and remote working are related to emerging immediate needs for employment security and food assistance (20).

For university students, their psychosocial needs under COVID-19 are closely related to adjustment to remote learning and the lack of a “normal social life” due to campus lockdown and other social distancing measures. Conceptually, there are two broad categories of such needs, including personal needs and interpersonal needs. Regarding personal needs, university students have academic needs, including a good study environment, learning strategies, and technology literacy to have effective online learning. Besides, they also need good physical fitness, emotional health, self-discipline, prevention of COVID-19, and sound financial condition to thrive. Furthermore, university students need to satisfy their interpersonal needs under lockdown, such as getting connected with friends and family members, having a sense of belonging to the university, and feeling safe in the community. In this study, we assessed the satisfaction of university students' specific psychosocial needs under COVID-19 based on the qualitative data collected in a previous study.

Besides needs, students also face difficulties and challenges (i.e., stressors), which might adversely affect their mental health. Primarily, personal and academic challenges are commonly encountered by university students under COVID-19, such as disruption of study, inhibited learning motivation, academic stress, difficulty in enjoying a normal university life such as joining internships, and worry about prospects. The second area of difficulties surrounds online learning, such as online study problems, computer technology problems, and group project difficulties. Finally, university students face physical and mental health difficulties, including physical symptoms, exercise problems, emotional symptoms, fear of going out, tiredness of staying at home, and COVID-19 fatigue. Theoretical models suggest that stressors and difficulties encountered in life (such as daily hassles) negatively contribute to mental health. Research also showed that higher daily hassles and stressors predicted poorer mental health (21, 22). While there are established general measures of stressors or hassles, few studies have examined difficulties under COVID-19. Hence, based on the findings of a focus group study, we constructed a scale to assess life difficulties faced by university students under COVID-19.

How might satisfaction of needs and life difficulties influence mental health? Based on COVID-19 studies on Chinese young people, Shek (15) argued the need to consider the intervening mechanisms involved. In this study, we proposed a model in which need satisfaction and difficulties encountered would predict an individual's stress symptoms, which would further shape mental health indexed by depression and anxiety. Primarily, this framework aligns with the notion that need satisfaction may serve as a coping resource (17), which helps university students better cope with challenging situations related to COVID-19 and reduces stress levels. Indeed, Li et al. (23) showed a negative relationship between need satisfaction and stress level among university athletes. Furthermore, life difficulties are sources of stress (i.e., daily stressors) that may further reinforce maladaptive responses and increase stress levels. In fact, a high level of stress is generally considered a threat to one's health and wellbeing (24). It has been reported to be a salient predictor of mental health problems among young people under COVID-19, including anxiety and depressive symptoms (25, 26).

Based on the above framework, we asked the following research questions in this study:

1. What are the prevalence rates of stress, depression, and anxiety among university students in Hong Kong?
2. What are the correlates of stress, depression, and anxiety in Hong Kong university students? We examined three correlates in this study. First, as Hong Kong students were stressed and the “social event” in 2019 was disturbing (13), we expected that stress level and emotional distress would be higher in local students than in international students (Hypotheses 1a, 1b, and 1c for stress, depression, and anxiety, respectively). Second, as social support is important under crisis (27), we expected that students living alone would display higher levels of stress, depression, and anxiety than those living with their family or roommates (Hypotheses 2a, 2b, and 2c, respectively). Third, as economic strain is a risk factor for youth mental health (28), we hypothesized that compared with those without economic strain, students experiencing economic strain would report higher levels of stress, depression, and anxiety (Hypothesis 3a, 3b, and 3c, respectively).
3. Is need satisfaction under COVID-19 related to mental health of the university students? Based on the literature (23), we hypothesized that need satisfaction would be negatively related to stress, depression, and anxiety (Hypotheses 4a, 4b, and 4c, respectively).
4. Are difficulties encountered by university students under COVID-19 related to their mental health? With reference to the literature (21, 22), we expected that there would be positive relationships between difficulties and stress, depression, and anxiety symptoms (Hypotheses 5a, 5b, and 5c, respectively).
5. Does stress mediate the relationships between need satisfaction and difficulties encountered and mental health measures? Based on the preceding discussion, we hypothesized that stress would mediate the influence of need satisfaction and difficulties encountered on mental health indexed by depression and anxiety (Hypothesis 6).

## Methods

### Participants and Procedures

Data were collected from undergraduate students at one university between January and March 2021 (i.e., after the fourth wave of the pandemic in Hong Kong). The total number of undergraduate students at this university was 15,271 as of the data collection period. A quota sampling strategy with faculty and study year as the stratifying factors was adopted. Instead of randomly selecting the students, we recruited subjects fulfilling different criteria for different groups. The approach is different from stratified random sampling because quota sampling represents non-random sampling. We used this method because there was difficulty in getting the full list of students. In addition, this approach is a more viable approach under COVID-19. Quota sampling was used in studies under COVID-19 (29–32), with some of them claiming that the samples were representative of the populations under investigation (30, 31).

As the campus was partially locked down and students attended lectures online during this period, we used an online questionnaire platform (Qualtrics XM) to collect data. The questionnaire was in English, which is the medium of instruction in this university. Participants were informed about the study purpose, confidential use of data, principles of voluntary participation, and free withdrawal before responding to the questionnaire. Informed consent was obtained from the participants. Participants who completed the questionnaire would receive a HK$100 (= US$12.8) supermarket voucher as an incentive. This project has received ethical approval from the institutional review board of the university.

To increase the data quality, we inserted two instructional manipulation check questions, in which the respondents were requested to select the instructed option (e.g., “This is an attention check, please choose exactly true”) to identify respondents who completed the survey carelessly (33, 34). Among 2,050 students who responded to the questionnaire, 12 students did not complete the consent form, 21 students were not full-time undergraduate students, and 369 students did not pass the “attention checking” test. As a result, we had 1,648 effective completed questionnaires (Mean age = 20.09 years ± 1.37).

### Instruments

#### Depression Anxiety Stress Scale (DASS-21)

The DASS-21 is a 21-item self-report scale assessing depression (7 items, e.g., “I couldn't seem to experience any positive feelings at all”), anxiety (7 items, e.g., “I was aware of dryness of my mouth”), and stress (7 items, e.g., “I found it hard to wind down”) (35). The DASS-21 has been validated using a sample of university students in Hong Kong (12), supporting the psychometric properties of the scale. Respondents indicated the frequency of experiencing each state over the past week on a 4-point Likert scale (0 = Not at all; 3 = Most of the time). The severity of depression, anxiety, and stress was classified into five categories, including normal, mild, moderate, severe, and extremely severe (35). The full scale and three subscales showed good reliability with Cronbach's alphas above 0.80 (see Table 1).

**Table 1:** Statistics of mean, SD, reliability, and correlations.

	**Variables**	**Mean (SD)**	**Cronbach's α** **(mean inter-item** **correlations)**	**1**	**2**	**3**	**4**	**5**	**6**	**7**	**8**	**9**	**10**
1	Age	20.09 (1.37)	–										
2	Gender^a^	–	–	−0.023									
3	Local/International^b^	–	–	0.126^***^	0.064^*^								
4	Living status^c^	–	–	0.089^***^	−0.033	0.159^***^							
5	Economic strain^d^	0.37 (0.48)	0.80(0.58)	0.054^*^	−0.013	−0.025	0.017						
6	Need satisfaction	3.78 (0.73)	0.89(0.34)	−0.047	0.065^*^	0.090^**^	−0.004	−0.107^***^					
7	Difficulties	3.10 (0.60)	0.91(0.30)	−0.007	0.104^***^	0.035	0.006	0.168^***^	−0.150^***^				
8	DASS-depression	5.91 (4.43)	0.88(0.52)	−0.025	−0.037	−0.072^**^	0.062^*^	0.192^***^	−0.222^***^	0.435^***^			
9	DASS-anxiety	5.39 (4.15)	0.86(0.46)	−0.078^**^	−0.045	−0.064^**^	0.091^***^	0.176^***^	−0.125^***^	0.413^***^	0.785^***^		
10	DASS-stress	6.27 (4.46)	0.89(0.54)	−0.053^*^	−0.037	−0.062^*^	0.084^***^	0.192^***^	−0.180^***^	0.456^***^	0.851^***^	0.859^***^	
11	DASS total	17.57 (12.29)	0.95(0.49)	−0.054^*^	−0.042	−0.070^**^	0.083^***^	0.198^***^	−0.188^***^	0.462^***^	0.934^***^	0.932^***^	0.960^***^

a *Male = 1, Female = 2.*

b *Local student = 1, International student = 2.*

c *Living with others = 1, Living alone = 2.*

d *Do not experience economic strain = 0, Experience economic strain = 1.*

* *p < 0.05;*

** *p < 0.01;*

*** *p < 0.001*.

#### Need Satisfaction Under COVID-19

Two pilot focus group interviews (*N* = 22 students) were conducted before the development of the online questionnaire to understand the needs of students during the pandemic. We identified 15 needs based on the findings, including physical (e.g., “prevent infection of COVID-19”), psychological (e.g., “keep good emotional health”), social (e.g., “make new friends at the university”), familial (e.g., “maintain harmony in family”), and academic needs (e.g., “have a good learning environment”). Respondents were asked to indicate how well these needs were met in the past year on a 6-point Likert scale, ranging from 1 (Not met at all) to 6 (Fully met). This scale had good reliability with Cronbach's alpha >0.80 (see Table 1).

#### Difficulties Encountered Under COVID-19

Twenty-four difficulties and challenges were identified from the focus group interviews and previous research on COVID-19, including physical (e.g., “dry eyes and back pain because of using computer for long hours”), psychological (e.g., “negative emotions and loneliness”), social (e.g., “hard to make new friends”), familial (e.g., “have conflicts with family members”), financial (e.g., “hard to do or get a part-time work”), academic (e.g., “low learning motivation”), online learning (e.g., “online learning platforms or systems do not work well on my devices”), university life (e.g., “not having a normal university life”), and career (e.g., “worry about future career”). Respondents indicated how often they encountered difficulties and challenges in the past year on a 5-point Likert scale ranging from 1 (Never) to 5 (Always). This scale demonstrated good reliability (Cronbach's alpha = 0.91, see Table 1).

The two newly developed scales on need satisfaction and difficulties showed good psychometric properties. The items were based on findings of student focus group interviews aiming to understand their needs and difficulties during the pandemic and thus were representative. In addition, the items were supported by the review and consensus of several researchers in adolescent development, which also supported the content validity of the measure (36). In addition, the results of reliability analyses showed that the items measured homogenous concepts and had good internal consistency. In the present study, we treated the scales as a whole without looking at the specific dimensions, which is not uncommon in social science research (37–40).

#### Economic Strain

We developed a composite measure of economic strain, which included three items on a dichotomous scale (yes/no): “does your family experience financial difficulty at the present time?,” “do you experience financial difficulty at the present time?” and “are you or your family member(s) become unemployed during the COVID-19 pandemic?” If students responded “no” to all three items, the final economic strain was coded as “0,” meaning “not experiencing economic strain.” Otherwise, it was coded as “1,” indicating “experiencing economic strain.” This scale had good reliability (Cronbach's alpha = 0.80, see Table 1).

### Data Analysis

We utilized SPSS 26.0 for data analysis. MANOVAs were used to explore the correlates of students' mental health with depression, anxiety, and stress as three inter-related dependent variables. We examined the overall difference (e.g., gender) on these dependent variables first (i.e., omnibus test). If the omnibus effect was significant, follow-up univariate tests were performed separately for each dependent variable. This procedure is commonly adopted when there are more than one dependent variable for a global measure in MANOVAs (41). Correlation analyses were performed to examine the relationships between need satisfaction, difficulties, and mental health. Mediation models were examined using PROCESS macro in SPSS.

## Results

The mean scores of stress, depression, and anxiety were 6.27, 5.91, and 5.39, respectively, based on DASS-21 (see Table 1). The scores were multiplied by 2 to calculate the final score for DASS-42 (see Table 2). According to the recommended cutoffs for moderate levels of morbidity for DASS-42 (35), the prevalence rates of moderate and above levels of stress, depression, and anxiety were 22.2% (95% CI 20.22–24.29), 40.0% (95% CI 37.67–42.46), and 50.7% (95% CI 48.28–53.17), respectively (see Table 2).

**Table 2:** Severity distribution of depression, anxiety, stress (*N* = 1,648).

**Domain**	**Mean (SD)** **DASS-21**	**Mean (SD)** **DASS-42**	**Normal** **count (%)**	**Mild** **count (%)**	**Moderate** **count (%)**	**Severe** **count (%)**	**Extremely severe** **count (%)**
Depression	5.91 (4.43)	11.81 (8.86)	736 (44.7%)	252 (15.3%)	377 (22.9%)	171 (10.4%)	112 (6.8%)
Anxiety	5.39 (4.15)	10.78 (8.3)	643 (39.0%)	169 (10.3%)	378 (22.9%)	175 (10.6%)	283 (17.2%)
Stress	6.27 (4.46)	12.55 (8.92)	1068 (64.8%)	214 (13.0%)	184 (11.2%)	151 (9.2%)	31 (1.9%)

*Cutoffs for Depression: Normal (0–9), Mild (10–13), Moderate (14–20), Severe (21–27), Extremely Severe (28+). Cutoffs for Anxiety: Normal (0–7), Mild (8–9), Moderate (10–14), Severe (15–19), Extremely Severe (20+). Cutoffs for Stress: Normal (0–14), Mild (15–18), Moderate (19–25), Severe (26–33), Extremely Severe (34+). The cutoffs are based on DASS-42*.

As shown in Table 3, results of MANOVAs showed that local students felt more depressed, anxious, and stressed than did international students, supporting Hypotheses 1a−1c. Students living alone demonstrated more depression, anxiety, and stress than did those living with others, which supported Hypotheses 2a−2c. Lastly, students experiencing economic strain displayed higher stress, anxiety, and depression levels than did those without economic strain. Hypotheses 3a−3c were supported.

**Table 3:** Results of MANOVA.

	**Valid *N* (%)**	**Mean (SD)**			**Omnibus effect**
		**Depression**	**Anxiety**	**Stress**	
Local/international					
*F*		8.559^**^	6.677^*^	6.336^*^	2.95^*^
*$\eta_{p}^{2}$*		0.005	0.004	0.004	0.005
Total	1648 (100.0%)	5.91 (4.43)	5.39 (4.15)	6.27 (4.46)	
Local student	1613 (97.9%)	5.95 (4.46)	5.43 (4.18)	6.31 (4.48)	
International student	35 (2.1%)	3.74 (2.21)	3.60 (2.03)	4.40 (2.52)	
Living status					
*F*		6.290^*^	13.734^***^	11.571^**^	5.01^**^
*$\eta_{p}^{2}$*		0.004	0.008	0.007	0.009
Total	1648 (100.0%)	5.91 (4.43)	5.39 (4.15)	6.27 (4.46)	
Live with others (family or roommates)	1615 (98.0%)	5.87 (4.42)	5.34 (4.12)	6.22 (4.42)	
Live alone	33 (2.0%)	7.82 (4.54)	8.03 (4.82)	8.88 (5.37)	
Economic strain					
*F*		61.94^***^	52.16^***^	62.15^***^	22.46^***^
*$\eta_{p}^{2}$*		0.037	0.031	0.037	0.040
Total	1629 (100.0%)	5.88 (4.41)	5.36 (4.13)	6.26 (4.45)	
Do not experience economic strain	1031 (63.3%)	5.24 (4.16)	4.81 (3.82)	5.61 (4.14)	
Experience economic strain	598 (36.7%)	6.99 (4.61)	6.32 (4.46)	7.38 (4.75)	

* *p < 0.05;*

** *p < 0.01;*

*** *p < 0.001*.

Students reported relatively low levels of satisfaction of needs for effective learning, a normal social life, and staying mentally and physically healthy (see Table 4). Students encountered the greatest difficulties in enjoying a normal university life (e.g., meeting with friends and having exchange or internship experiences) and difficulties due to lockdown or social distancing (e.g., tired of staying at home and difficult to workout, see Table 4).

**Table 4:** Response profiles of need satisfaction and difficulties.

	**Negative response**		**Positive response**	
	***N***	**%**	***N***	**%**
**Need satisfaction** ^**a**^				
1. Prevent infection of COVID-19	344	20.87	1,304	79.13
2. Keep physical fitness	639	**38.77**	1,009	61.23
3. Keep good emotional health	525	**31.86**	1,123	68.14
4. Maintain a sound financial condition	543	**32.95**	1,105	67.05
5. Have a good learning environment	461	27.97	1,187	72.03
6. Have effective online learning strategy	555	**33.68**	1,093	66.32
7. Have self-discipline	640	**38.83**	1,008	61.17
8. Have technology literacy	408	24.76	1,240	75.24
9. Make new friends at the University	878	**53.28**	770	46.72
10. Go out with friends	668	**40.53**	980	59.47
11. Maintain close connection with friends	559	**33.92**	1,089	66.08
12. Maintain harmony in family	445	27.00	1,203	73.00
13. Feel safe and relax in community	616	**37.38**	1,032	62.62
14. Have a sense of connection or belonging to the University	882	**53.52**	766	46.48
15. Get comprehensive and consistent guideline from the University	839	**50.91**	809	49.09
16. Make new friends at the University	878	**53.28**	770	46.72
**Difficulties** ^**b**^				
1. Physical symptoms because of using computer for long hours	433	26.27	1,215	**73.73**
2. Difficult to exercise	337	20.45	1,311	**79.55**
3. Emotional symptoms	524	31.80	1,124	68.20
4. Tired of staying at home	461	27.97	1,187	**72.03**
5. Afraid of going out	647	39.26	1,001	60.74
6. COVID-19 fatigue	474	28.76	1,174	**71.24**
7. Hard to do or get a part-time work	479	29.07	1,169	**70.93**
8. Lack of technology literacy	799	48.48	849	51.52
9. The online learning platforms or systems do not work well on my devices	733	44.48	915	55.52
10. Encounter connection problems during online lecture or assessment	722	43.81	926	56.19
11. Easily distracted during online lectures	328	19.90	1,320	**80.10**
12. Low learning motivation	277	16.81	1,371	**83.19**
13. Lack of effective online learning strategy	355	21.54	1,293	**78.46**
14. Hard to find time and a place to meet groupmates when doing group project	479	29.07	1,169	**70.93**
15. Online communication issues with groupmates	436	26.46	1,212	**73.54**
16. Free-rider issue in group project	699	42.42	949	57.58
17. Worry about academic performance	275	16.69	1,373	**83.31**
18. Have conflicts with family members	773	46.91	875	53.09
19. Competition on learning resources and space in family	830	50.36	818	49.64
20. Hard to make new friends	316	19.17	1,332	**80.83**
21. Hard to meet friends face to face	247	14.99	1,401	**85.01**
22. Do not have a normal university life	201	12.20	1,447	**87.80**
23. Hard to do an internship or go on exchange	267	16.20	1,381	**83.80**
24. Worry about future career	284	17.23	1,364	**82.77**

*Need satisfaction: values in the “Negative response” column greater than 30% are bold, meaning that at least 30% of the participants indicated that their specific needs were not met. Difficulties: values in the “Positive response” column greater than 70% are bold, meaning that more than 70% of the participants experienced difficulties in specific areas.*

a *Need satisfaction uses a 6-point Likert scale. Negative responses include options 1 = Not met at all, 2 = Moderately not met, and 3 = Slightly not met. Positive responses contain options 4 = Slightly met, 5 = Moderately met, and 6 = Fully met. Negative response rates above 30% are bolded.*

b *Difficulties use a 5-point Likert scale. Negative responses include options 1 (Never) and 2 (Rarely). Positive responses include options 3 (Sometimes), 4 (Often), and 5 (Always). Positive response rates above 70% are bolded*.

**Figure 1:**
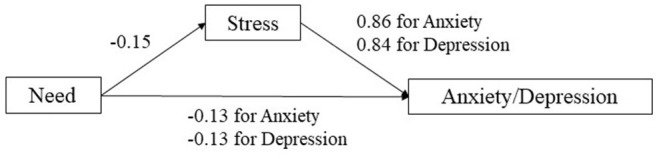
Effects of need satisfaction on anxiety and depression with stress as the mediator.

**Figure 2:**
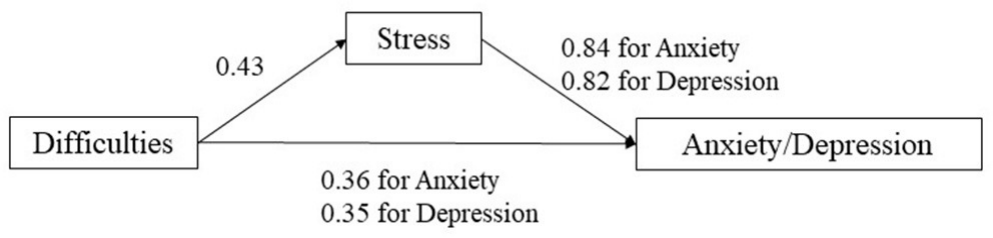
Effects of difficulties on anxiety and depression with stress as the mediator.

Table 1 shows that need satisfaction was negatively correlated with stress (*r* = −0.180), depression (*r* = −0.222), and anxiety (*r* = −0.125), which supported Hypotheses 4a−4c. Besides, difficulties were positively related to stress (*r* = 0.456), depression (*r* = 0.435), and anxiety (*r* = 0.413). Hypotheses 5a−5c were also supported. Table 5 summarizes the results of regression analyses examining the role of stress in mediating the relationships between need satisfaction and difficulties and mental health (i.e., depression and anxiety). Age, gender, local/international student, living status, and economic strain were controlled in all models (see full results in Appendices 1–4 in Supplementary Material). In line with the hypotheses, stress significantly mediated the relationships between need satisfaction and difficulties and mental health (see Figures 1, 2). Specifically, stress showed a full mediation effect in the relationship between need satisfaction and anxiety, as the direct effect of need satisfaction on anxiety became non-significant when the mediator (stress) was added. For other mediation models, partial mediation effects were observed (see Table 5). Therefore, Hypothesis 6 was also supported.

**Table 5:** Mediating effects of stress (Mediator) for the effect of need satisfaction and difficulties (IV) on anxiety and depression (DV).

**Regression model for anxiety (DV)**	**Need satisfaction (IV)**			**Difficulties (IV)**		
	**β**	***SE***	***t***	**β**	***SE***	***t***
Total effect of IV on DV (anxiety)	−0.11	0.14	−4.26^***^	0.39	0.16	16.68^***^
IV to Mediator	−0.15	0.15	−6.07^***^	0.43	0.17	18.59^***^
Mediator to DV (anxiety)	0.86	0.01	63.42^***^	0.84	0.01	56.77^***^
Direct effect of IV on DV (anxiety)	0.02	0.08	1.73	0.03	0.1	2.14^*^
**Mediating effect**	**Point estimate**	**Bootstrapping** **(BC 95% CI)**		**Point estimate**	**Bootstrapping** **(BC 95% CI)**	
		**Lower**	**Upper**		**Lower**	**Lower**
	−0.13	−0.18	−0.08	0.36	0.33	0.40
**Regression model for depression (DV)**	**Need satisfaction (IV)**			**Difficulties (IV)**		
	***β***	***SE***	***t***	***β***	***SE***	***t***
Total effect of IV on DV (depression)	−0.18	0.15	−7.34^***^	0.41	0.17	17.41^***^
IV to Mediator	−0.15	0.15	−6.07^***^	0.43	0.17	18.59^***^
Mediator to DV (depression)	0.84	0.01	60.88^***^	0.82	0.01	54.57^***^
Direct effect of IV on DV (depression)	−0.06	0.08	−4.08^***^	0.05	0.11	3.53^***^
**Mediating effect**	**Point estimate**	**Bootstrapping** **(BC 95% CI)**		**Point estimate**	**Bootstrapping** **(BC 95% CI)**	
		**Lower**	**Upper**		**Lower**	**Lower**
	−0.13	−0.17	−0.08	0.35	0.32	0.39

* *p < 0.05;*

*** *p < 0.001*.

## Discussion

The present study investigated stress, depression, and anxiety as well as the related correlates among university students in Hong Kong under COVID-19. We also examined their psychosocial need satisfaction and difficulties in daily lives and how these factors predicted mental health indexed by depression and anxiety through the mediating effect of stress responses. This study was conducted roughly one year after the first large-scale outbreak of COVID-19 in Wuhan, China, in December 2019. In Hong Kong, there had been four waves of outbreaks when this study was conducted. Using the different phases of stress response as a reference, Hong Kong had passed the “alarm” phase and adjusted in the “resistance” phase. Given this unique background, the current findings have both theoretical and practical significance.

Regarding the prevalence rates of mental health issues among university students, the findings showed that 22.2, 50.7, and 40.0% of the respondents in Hong Kong could be classified as showing at least moderate levels of stress, anxiety, and depression, respectively. The figures were relatively higher than those among Hong Kong university students before COVID-19 (12), which showed 18.5, 44.7, and 32.7% as the corresponding figures for these three measures, respectively. However, the figures of the present study were lower than or equivalent to other findings during COVID-19 (26, 42). For example, Varma et al. (26) reported that 77, 59, and 35% of adult respondents from 63 countries (e.g., Australia, India, and the United Kingdom) reported moderate or greater levels of stress, anxiety, and depression, respectively. In addition, a study involving 1,210 respondents from 194 cities in Mainland China found over half of the respondents had experienced moderate to severe negative psychological impacts of COVID-19 (43), with around 29% reported moderate to severe anxiety symptoms and 17% reported moderate to severe depressive symptoms. In a recent study in Brazil, the prevalence rates were 45, 46.3, and 48.7% of the students were classified as having at least moderate levels of stress, anxiety, and depression, respectively (44). These comparisons suggested that while COVID-19 had negatively impacted Hong Kong university students' mental health, yet students might have adjusted to the “new normal life” under COVID-19 after four waves of the pandemic when the data were collected. In addition, the university had stepped up measures (e.g., student counseling) to support students, which might counteract the negative impacts. In fact, another recent study showed that the prevalence of post-traumatic stress disorder (PTSD) after one year of the onset of COVID-19 was 12.4%, which was lower than the previous studies conducted at the early stage of the pandemic (45). These findings suggest that we have to understand the impact of COVID-19 with reference to different waves of the pandemic. Obviously, with the rapid onset of the fifth wave due to Omicron in Hong Kong (since early January 2022), the mental health of university students may be adversely affected because many people died in the recent Omicron outbreak.

For the correlates of negative mental states, the findings are generally in line with the original expectations that being a local student, living alone, and experiencing economic strain would be associated with higher levels of negative mental health (Hypotheses 1a−1c, 2a−2c, and 3a−3c). However, it is surprising that being a local student seems to be a risk factor because international students are believed to have poorer mental health due to travel restrictions. As we have argued, one possible contributing factor is the joint influence of social unrest and COVID-19 in the 2019–2020 academic year, which are very traumatic to the local university students (13). Nevertheless, as the numbers of international students and those living alone were limited, more studies are needed to replicate the present findings.

Regarding need satisfaction, there are concerns on learning, social, and health domains. These findings represent more “specific” needs and challenges associated with online learning and social distancing measures under COVID-19 instead of “general” needs and stressors covered in the existing COVID-19 studies. Similarly, responses to the difficulties items also showed that study, interpersonal relationships, and health were major concerns. Obviously, measures should be devised to meet the needs and help students cope with challenges. Shek et al. (46) argued that positive youth development programs would help to build up “inner strengths” of young people, which allow students to cope effectively under the influences of stress.

Consistent with our hypotheses, need satisfaction and life difficulties were predictors of mental health (Hypotheses 4a−4c and 5a−5c), and perceived stress served as a mediator of such prediction effects (Hypothesis 6). While the roles of general psychological need satisfaction and life challenges in mental health have been well-established (17, 18, 21), the present study further expands the scope of such influence by focusing on immediate psychosocial needs and challenges under COVID-19. It can be argued that such “specific” needs and difficulties represent elements in daily lives that underline the basic psychological needs during COVID-19. For example, items related to online learning are arguably related to the need for competence, while those social-relational items are associated with the need for relatedness. In addition, the present study highlights the mediating role of stress response in linking need satisfaction and difficulties and mental health, which is in line with previous findings (23–26). Students' need satisfaction could serve as a coping resource reducing their stressed feelings, which consequently promotes their mental health under the COVID-19. Besides, Hu et al. (47) also showed that perceived stress mediated the impact of hope on post-stress growth. On the other hand, students' perceived difficulties would increase their stress levels, hence jeopardizing their mental health and wellbeing.

Practically speaking, universities need to provide services (e.g., support or counseling) to address the specific needs and difficulties experienced by students to improve their mental health status. At the same time, it is also important to equip students with sufficient skills to handle difficulties and cope with stress effectively (16, 46).

Despite the pioneering nature of this study in Hong Kong, there are several limitations of this study. First, although the sample size is large, this study only recruited students from one university. Second, this study used quota sampling design with faculty and year as the stratifying factors (i.e., dimensions that formed different groups) instead of a stratified random sampling design. As suggested by Iliyasu and Etikan (48), using quota sampling may have limitations such as the probability of biased collection of respondents based on invalid quota controls, although it also has the advantages of ease and efficiency and it could be practically used when it is difficult to obtain the population list and engage the potential participants under COVID-19. It is noteworthy that some studies showed that the effectiveness of random sampling was higher than that of quota sampling (49). Third, this is a cross-sectional study only. It would be helpful if longitudinal studies could be conducted in future. Fourth, as the study was conducted at the “resistance” and/or “exhaustion” phase after four outbreaks, the findings may be different from those arising from other phases of the pandemic. Finally, the correlation coefficients for relationships between socio-demographic variables and DASS showed small effect sizes, which should be interpreted with caution. Although statistical significance showed the effects of these variables, practical significance may suggest that the effects were not large for practical outcomes, particularly for studies with large sample sizes. However, we should note three points here. First, in social science research, the magnitude of correlations is usually not high (50). Second, the low correlations were mainly confined to those between socio-demographic variables and DASS measures. For the correlations between needs satisfaction (or difficulties) and DASS measures, the values were of moderate magnitude. Third, low correlation coefficients on the relationships between socio-demographic variables and mental health indicators were also reported in similar studies (51). According to Bakker et al. (52), examining effect size with similar benchmark studies is important. Despite these limitations, the present study contributes to our understanding of student mental health roughly one year after the outbreak of COVID-19 in December 2019.

## Data Availability Statement

The raw data supporting the conclusions of this article will be made available by the authors, without undue reservation.

## Ethics Statement

The studies involving human participants were reviewed and approved by Institutional Review Board of the Hong Kong Polytechnic University. The patients/participants provided their written informed consent to participate in this study.

## Author Contributions

DS, DD, and XZ: conceptualization, methodology, writing—original draft preparation, writing—review and editing, data collection, revision, and editing. DD: formal analysis. DS and XZ: supervision. DS: project administration and funding acquisition. All authors have read and agreed to the published version of the manuscript.

## Funding

This project and this paper are financially supported by a UGC special grant for student support services in response to the COVID-19 pandemic entitled Promotion of Psychological Wellbeing in University Students under COVID-19: Needs assessment and mental health survey (Project No. 89P9).

## Conflict of Interest

The authors declare that the research was conducted in the absence of any commercial or financial relationships that could be construed as a potential conflict of interest.

## Publisher's Note

All claims expressed in this article are solely those of the authors and do not necessarily represent those of their affiliated organizations, or those of the publisher, the editors and the reviewers. Any product that may be evaluated in this article, or claim that may be made by its manufacturer, is not guaranteed or endorsed by the publisher.
