# Supplementary material for: Prevalence and Correlates of Mental Health of University Students in Hong Kong: What Happened One Year After the Occurrence of COVID-19?
Source: Front Public Health. 2022 Jun 29;10:857147. doi: 10.3389/fpubh.2022.857147 (PMC9277093; doi:10.3389/fpubh.2022.857147)
Supplement: Supplementary file 1 [file Table_1.DOCX]

Appendix 1. Mediating effects of stress (Mediator) for the effect of need satisfaction (IV) on anxiety (DV)

|  | *β* | SE | *t* | B | 95% CI | |
| --- | --- | --- | --- | --- | --- | --- |
|  |  |  |  |  | Lower | Upper |
| **Direct effects of IV on Mediator** |  |  |  |  |  |  |
| Need | -0.15 | 0.15 | -6.07*** | -0.93 | -1.23 | -0.63 |
| Age | -0.06 | 0.08 | -2.51* | -0.20 | -0.36 | -0.04 |
| Gender^a^ | -0.01 | 0.22 | -0.54 | -0.12 | -0.55 | 0.31 |
| Local/International^b^ | -0.05 | 0.76 | -1.78 | -1.35 | -2.83 | 0.14 |
| Living status^c^ | 0.06 | 0.82 | 2.26* | 1.86 | 0.25 | 3.48 |
| Economic strain^d^ | 0.19 | 0.23 | 7.77*** | 1.77 | 1.32 | 2.21 |
| **Direct effects of IV and Mediator on DV** |  |  |  |  |  |  |
| Need | 0.02 | 0.08 | 1.73 | 0.13 | -0.02 | 0.28 |
| Stess | 0.86 | 0.01 | 63.42*** | 0.80 | 0.77 | 0.82 |
| Age | -0.04 | 0.04 | -3.12*** | -0.12 | -0.20 | -0.05 |
| Gender^a^ | -0.01 | 0.11 | -1.04 | -0.11 | -0.32 | 0.10 |
| Local/International^b^ | -0.01 | 0.37 | -0.58 | -0.22 | -0.94 | 0.51 |
| Living status^c^ | 0.02 | 0.40 | 1.73 | 0.70 | -0.09 | 1.49 |
| Economic strain^d^ | 0.02 | 0.11 | 1.30 | 0.15 | -0.07 | 0.37 |
| **Total effects of IV on DV** |  |  |  |  |  |  |
| Need | -0.11 | 0.14 | -4.26*** | -0.61 | -0.89 | -0.33 |
| Age | -0.10 | 0.08 | -3.77*** | -0.29 | -0.44 | -0.14 |
| Gender^a^ | -0.03 | 0.20 | -1.00 | -0.21 | -0.61 | 0.20 |
| Local/International^b^ | -0.05 | 0.71 | -1.82 | -1.29 | -2.68 | 0.10 |
| Living status^c^ | 0.07 | 0.77 | 2.84*** | 2.18 | 0.67 | 3.69 |
| Economic strain^d^ | 0.18 | 0.21 | 7.32*** | 1.55 | 1.14 | 1.97 |

^a^ Male=1, Female=2. ^b^ Local student=1, International student=2. ^c^ Living with others = 1, Living alone =2. ^d^ Do not experience economic strain = 0, Experience economic strain = 1. * *p* < .05; *** *p* < .001.

Appendix 2. Mediating effects of stress (Mediator) for the effect of need satisfaction (IV) on depression (DV)

|  | *β* | SE | *t* | B | 95% CI | |
| --- | --- | --- | --- | --- | --- | --- |
|  |  |  |  |  | Lower | Upper |
| **Direct effects of IV on Mediator** |  |  |  |  |  |  |
| Need | -0.15 | 0.15 | -6.07*** | -0.93 | -1.23 | -0.63 |
| Age | -0.06 | 0.08 | -2.51* | -0.20 | -0.36 | -0.04 |
| Gender^a^ | -0.01 | 0.22 | -0.54 | -0.12 | -0.55 | 0.31 |
| Local/International^b^ | -0.05 | 0.76 | -1.78 | -1.35 | -2.83 | 0.14 |
| Living status^c^ | 0.06 | 0.82 | 2.26* | 1.86 | 0.25 | 3.48 |
| Economic strain^d^ | 0.19 | 0.23 | 7.77*** | 1.77 | 1.32 | 2.21 |
| **Direct effects of IV and Mediator on DV** |  |  |  |  |  |  |
| Need | -0.06 | 0.08 | -4.08*** | -0.33 | -0.49 | -0.17 |
| Stess | 0.84 | 0.01 | 60.88*** | 0.83 | 0.80 | 0.86 |
| Age | 0.02 | 0.04 | 1.25 | 0.05 | -0.03 | 0.14 |
| Gender^a^ | 0.00 | 0.12 | -0.12 | -0.01 | -0.24 | 0.21 |
| Local/International^b^ | -0.02 | 0.40 | -1.18 | -0.48 | -1.26 | 0.31 |
| Living status^c^ | 0.00 | 0.44 | -0.16 | -0.07 | -0.93 | 0.79 |
| Economic strain^d^ | 0.03 | 0.12 | 1.98 | 0.24 | 0.00 | 0.48 |
| **Total effects of IV on DV** |  |  |  |  |  |  |
| Need | -0.18 | 0.15 | -7.34*** | -1.10 | -1.40 | -0.81 |
| Age | -0.04 | 0.08 | -1.44 | -0.12 | -0.27 | 0.04 |
| Gender^a^ | -0.01 | 0.22 | -0.52 | -0.11 | -0.54 | 0.31 |
| Local/International^b^ | -0.05 | 0.75 | -2.13* | -1.59 | -3.06 | -0.13 |
| Living status^c^ | 0.05 | 0.81 | 1.82 | 1.48 | -0.11 | 3.07 |
| Economic strain^d^ | 0.19 | 0.22 | 7.64*** | 1.71 | 1.27 | 2.15 |

^a^ Male=1, Female=2. ^b^ Local student=1, International student=2. ^c^ Living with others = 1, Living alone =2. ^d^ Do not experience economic strain = 0, Experience economic strain = 1. * *p* < .05; *** *p* < .001.

Appendix 3. Mediating effects of stress (Mediator) for the effect of difficulties (IV) on anxiety (DV)

|  | *β* | SE | *t* | B | 95% CI | |
| --- | --- | --- | --- | --- | --- | --- |
|  |  |  |  |  | Lower | Upper |
| **Direct effects of IV on Mediator** |  |  |  |  |  |  |
| Difficulties | 0.43 | 0.17 | 18.59*** | 3.21 | 2.87 | 3.54 |
| Age | -0.05 | 0.07 | -2.33* | -0.17 | -0.32 | -0.03 |
| Gender^a^ | -0.07 | 0.20 | -2.96*** | -0.60 | -0.99 | -0.20 |
| Local/International^b^ | -0.08 | 0.69 | -3.26*** | -2.25 | -3.60 | -0.90 |
| Living status^c^ | 0.07 | 0.75 | 2.84*** | 2.13 | 0.66 | 3.60 |
| Economic strain^d^ | 0.13 | 0.21 | 5.51*** | 1.16 | 0.75 | 1.57 |
| **Direct effects of IV and Mediator on DV** |  |  |  |  |  |  |
| Difficulties | 0.03 | 0.10 | 2.14* | 0.22 | 0.02 | 0.42 |
| Stess | 0.84 | 0.01 | 56.77*** | 0.78 | 0.75 | 0.81 |
| Age | -0.04 | 0.04 | -3.33*** | -0.13 | -0.21 | -0.05 |
| Gender^a^ | -0.02 | 0.11 | -1.22 | -0.13 | -0.34 | 0.08 |
| Local/International^b^ | -0.01 | 0.37 | -0.55 | -0.20 | -0.93 | 0.52 |
| Living status^c^ | 0.02 | 0.40 | 1.81 | 0.73 | -0.06 | 1.52 |
| Economic strain^d^ | 0.01 | 0.11 | 0.95 | 0.11 | -0.11 | 0.33 |
| **Total effects of IV on DV** |  |  |  |  |  |  |
| Difficulties | 0.39 | 0.16 | 16.68*** | 2.72 | 2.40 | 3.04 |
| Age | -0.09 | 0.07 | -3.81*** | -0.27 | -0.40 | -0.13 |
| Gender^a^ | -0.07 | 0.19 | -3.14*** | -0.60 | -0.97 | -0.22 |
| Local/International^b^ | -0.07 | 0.65 | -3.00*** | -1.95 | -3.23 | -0.68 |
| Living status^c^ | 0.08 | 0.71 | 3.37*** | 2.39 | 1.00 | 3.78 |
| Economic strain^d^ | 0.12 | 0.20 | 5.09*** | 1.01 | 0.62 | 1.40 |

^a^ Male=1, Female=2. ^b^ Local student=1, International student=2. ^c^ Living with others = 1, Living alone =2. ^d^ Do not experience economic strain = 0, Experience economic strain = 1. * *p* < .05; *** *p* < .001.

Appendix 4. Mediating effects of stress (Mediator) for the effect of difficulties (IV) on depression (DV)

|  | *β* | SE | *t* | B | 95% CI | |
| --- | --- | --- | --- | --- | --- | --- |
|  |  |  |  |  | Lower | Upper |
| **Direct effects of IV on Mediator** |  |  |  |  |  |  |
| Difficulties | 0.43 | 0.17 | 18.59*** | 3.21 | 2.87 | 3.54 |
| Age | -0.05 | 0.07 | -2.33* | -0.17 | -0.32 | -0.03 |
| Gender^a^ | -0.07 | 0.20 | -2.96*** | -0.60 | -0.99 | -0.20 |
| Local/International^b^ | -0.08 | 0.69 | -3.26*** | -2.25 | -3.60 | -0.90 |
| Living status^c^ | 0.07 | 0.75 | 2.84*** | 2.13 | 0.66 | 3.60 |
| Economic strain^d^ | 0.13 | 0.21 | 5.51*** | 1.16 | 0.75 | 1.57 |
| **Direct effects of IV and Mediator on DV** |  |  |  |  |  |  |
| Difficulties | 0.05 | 0.11 | 3.53*** | 0.39 | 0.17 | 0.61 |
| Stess | 0.82 | 0.01 | 54.57*** | 0.82 | 0.79 | 0.84 |
| Age | 0.02 | 0.04 | 1.48 | 0.06 | -0.02 | 0.15 |
| Gender^a^ | -0.01 | 0.12 | -0.79 | -0.09 | -0.32 | 0.14 |
| Local/International^b^ | -0.02 | 0.40 | -1.82 | -0.73 | -1.52 | 0.06 |
| Living status^c^ | 0.00 | 0.44 | 0.02 | 0.01 | -0.85 | 0.87 |
| Economic strain^d^ | 0.03 | 0.12 | 1.85 | 0.23 | -0.01 | 0.47 |
| **Total effects of IV on DV** |  |  |  |  |  |  |
| Difficulties | 0.41 | 0.17 | 17.41*** | 3.01 | 2.67 | 3.35 |
| Age | -0.02 | 0.07 | -1.03 | -0.08 | -0.22 | 0.07 |
| Gender^a^ | -0.07 | 0.20 | -2.88*** | -0.58 | -0.98 | -0.18 |
| Local/International^b^ | -0.09 | 0.69 | -3.71*** | -2.56 | -3.91 | -1.21 |
| Living status^c^ | 0.05 | 0.75 | 2.32* | 1.75 | 0.27 | 3.22 |
| Economic strain^d^ | 0.13 | 0.21 | 5.57*** | 1.17 | 0.76 | 1.59 |

^a^ Male=1, Female=2. ^b^ Local student=1, International student=2. ^c^ Living with others = 1, Living alone =2. ^d^ Do not experience economic strain = 0, Experience economic strain = 1. * *p* < .05; *** *p* < .001
